# Inversion of the Patient

**Published:** 1863-08

**Authors:** 


*Inversion of the Patient.*—The taxis and every other means of reducing hernia failing, procure a board of five or six feet in length, and place and fix the patient by straps with his knees flexed over one end of the board. Then invert completely, or nearly so. The tumor will in many cases disappear spontaneously, and in very few will it refuse to return by a little manipulation. The explanation is the traction on the tumor exercised by the weight of the intestines, which of course gravitate from the tumor.—*Id.*

